# The Novel Parallel Closure Technique Compared to Single-Layer Closure of the Uterus After Primary Cesarean Section Decreases the Incidence of Isthmocele Formation and Increases Residual Myometrial Thickness

**DOI:** 10.7759/cureus.60932

**Published:** 2024-05-23

**Authors:** Ebru Alper, Ece Aksakal, Irem Usta, Bulent Urman

**Affiliations:** 1 Obstetrics and Gynecology, American Hospital, Istanbul, TUR

**Keywords:** implantation failure, vaginal birth after cesarean section, uterine niche, isthmocele, cesarean scar, cesarean section

## Abstract

Background

Isthmocele or a scar defect is a relatively common consequence of cesarean section resulting in menstrual disturbances and infertility and may compromise the myometrial integrity of the uterus in women contemplating subsequent vaginal birth. Several preventive measures have been suggested, including the modification of surgical techniques used for the closure of the uterine incision. The current study aimed to compare the incidence of isthmocele and assess residual myometrial thickness in women who underwent single versus parallel layered closure to approximate the endo-myometrial layer during cesarean section.

Methodology

This retrospective study evaluated data of women undergoing their first cesarean section under elective conditions (n = 497) where the uterine incision was closed using a single (n = 295) or a parallel layer (n = 202) technique. Patients were evaluated twice, at 3-6 months and 18 months postpartum, with a transvaginal ultrasound noting the presence or absence of an isthmocele and measurement of the residual myometrial thickness.

Results

Regardless of the closure technique, 64 (12.9%) women had an ultrasound-diagnosed isthmocele. Significantly fewer patients in the parallel-layer closure group presented with an isthmocele both at 3-6 (13.6 vs. 6.9%; p = 0.019) and 18 months (16.3 vs. 7.8%; p = 0.009) postpartum. Residual myometrium was significantly thicker in the parallel-layer closure group (8.0 vs. 13.2 mm at 3-6 months postpartum; p = 0.000 and 7.2 vs. 12.3 mm at 18 months postpartum; p = 0.004). For all patients, a retroverted position of the uterus at 3-6 months follow-up examination significantly increased the frequency of isthmocele (36/395 (9.1%) with an anteverted uterus and 18/102 (17.6%) with a retroverted uterus; p = 0.002). In patients with a single-layer closure, a retroverted uterus at the 3-6-month follow-up was associated with an isthmocele in 29.5% (18/61) of patients, while no isthmocele was recorded when the uterus was retroverted in the parallel-layer closure group (0/41) (p = 0.001). At 18 months postpartum, of the 64 patients with an isthmocele, 26 (40.6%) presented with abnormal uterine bleeding mainly in the form of postmenstrual spotting. Of the 26 patients with abnormal bleeding, 23 were in the single-layer and three were in the parallel-layer closure group.

Conclusions

The parallel-layer closure when compared to a single-layer closure of the uterine incision in patients undergoing primary cesarean section decreased the incidence of isthmocele formation and increased residual myometrial thickness. More patients in the single-layer closure group had menstrual cycle disturbances at 18 months postpartum.

## Introduction

Cesarean section rates are increasing globally, therefore, it is logical to expect more immediate and remote complications related to this procedure [1]. Isthmocele is a niche or a surgical scar defect that occurs following inappropriate healing of the lower uterine segment following cesarean section. The European Niche Taskforce defines isthmocele as an “indentation of the uterine myometrium of at least 2 mm at the site of the cesarean section scar,” also known as a uterine niche, uterine diverticulum, or cesarean scar defect [2]. It may result in obstetrical complications, such as cesarean scar pregnancy, uterine rupture, and placenta accreta spectrum disorders, gynecological symptoms mainly in the form of abnormal uterine bleeding (dysmenorrhea, pre and postmenstrual spotting, heavy or prolonged menses), and may impede a trial of labor in women considering vaginal birth after cesarean section [3,4]. Isthmoceles may be the cause of pelvic pain and secondary infertility [5]. Isthmocele, not cesarean section per se, also reduces the success of in vitro fertilization (IVF) in women presenting with infertility [6].

The technique of uterine closure during cesarean section has been implicated in the formation and severity of post-cesarean scar defects. Patient-related factors such as age, body mass index (BMI), prior cesarean section, presence of labor before cesarean section, the position of the uterus; labor-related factors such as dilatation, augmentation, premature rupture of membranes, and fetal station at cesarean section; and surgery-related factors such as surgical experience, operation time, choice of suture materials, running versus locking sutures, single versus double-layer closure of the myometrium, and inclusion versus exclusion of the endometrial layer have been studied [4].

While patient and labor-related factors cannot be affected, modification of surgical factors may have a beneficial impact on the prevalence and extent of cesarean scar defects with potential minimization of subsequent complications. Modifiable surgical factors include the choice of suture material and the method of incision closure. Synthetic absorbable sutures used in a nonlocking manner are preferred over catgut and locking sutures [7,8]. The inclusion or exclusion of the endometrial layer is controversial regarding isthmocele formation and residual thickness; however, the inclusion of the endometrial layer appeared to be more beneficial [9]. Studies are split over single versus double closure of the myometrial layer [4]. A recent randomized double-blind study performed among 2,292 women undergoing cesarean section failed to show any benefit of double-layer closure in terms of isthmocele development and postmenstrual spotting [10]. The study included patients who underwent cesarean section with and without prior labor and at different stages of cervical dilatation. Investigators of this study also examined the three-year gynecologic, fertility and obstetrical outcomes of the study cohort and failed to show any differences regarding live birth rates, need for fertility treatments, mode of delivery, or uterine ruptures in subsequent pregnancies [11]. Although double-layer closure may be associated with a better approximation of the incision, single-layer closure has the potential advantages of shorter operation time and less devascularization of the myometrial edges. The technique of double-layer closure varies from study to study. Different techniques have been defined such as interrupted second layer sutures, Z-sutures in the corners combined with interrupted sutures, far-far-near-near technique, and purse string closure. These methods have not been compared; however, the majority of the studies showed a decreased incidence of isthmocele and thicker residual myometrium when compared with a single-layer closure.

This study aimed to compare the incidence of isthmocele and thickness of the residual myometrium in women who underwent cesarean section in two consecutive periods initially with a single layer and subsequently with a parallel-layered closure of the lower uterine segment transverse incision.

## Materials and methods

**Figure 1:**
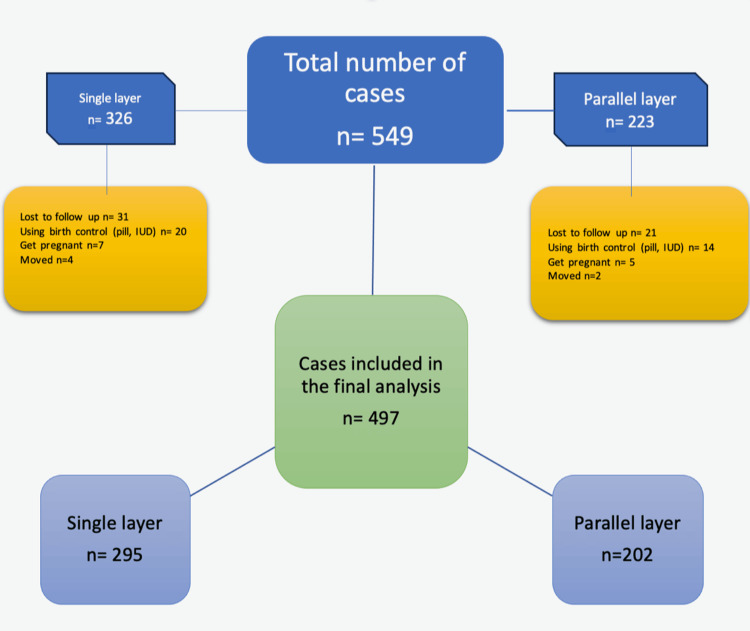
Flowchart of the study.

In this retrospective study, a total of 549 patients (326 in the single-layer and 223 in the parallel-layer group) who underwent cesarean section at the American Hospital, Women’s Health Clinic between March 2016 and December 2021 were included. Approval for the evaluation and publication of the collected data was obtained from the Koc University Committee for Human Research (approval number: 2015.019.IRB1.005). Only patients undergoing their first cesarean section under elective conditions were included. Cesarean sections performed upon patient request were also included in the group. In total, 52 patients (31 in the single-layer and 21 in the parallel-layer group) who were lost to follow-up, did not complete the follow-up, or were using contraceptive methods at the time of long-term evaluation were excluded. The data of 497 patients were included in the final analysis (Figure 1). After the exclusion of the patients who did not complete follow-up, 295 patients who underwent single-layer closure were compared with 202 patients who underwent parallel-layer closure.

All cesarean sections were performed by two surgeons using the same technique. Single-layer closure was favored in the initial phase of the study. Following the publication of Guidelines for Intraoperative Care in Cesarean Delivery by the Enhanced Recovery After Surgery Society in 2018 that concluded closure of the hysterotomy in two layers may be associated with a lower rate of uterine rupture (evidence level: low/ recommendation grade: weak), a decision was made to switch to parallel-layer closure in all patients [12].

The techniques were briefly as follows: single-layer closures were applied using 1 Vicryl sutures in a nonlocking manner. The endometrial layer was included in the suture line. Additional 00 Vicryl sutures were placed when necessary to control bleeding from the incision line. Visceral peritoneum was left open but the parietal peritoneum was closed.

**Figure 2:**
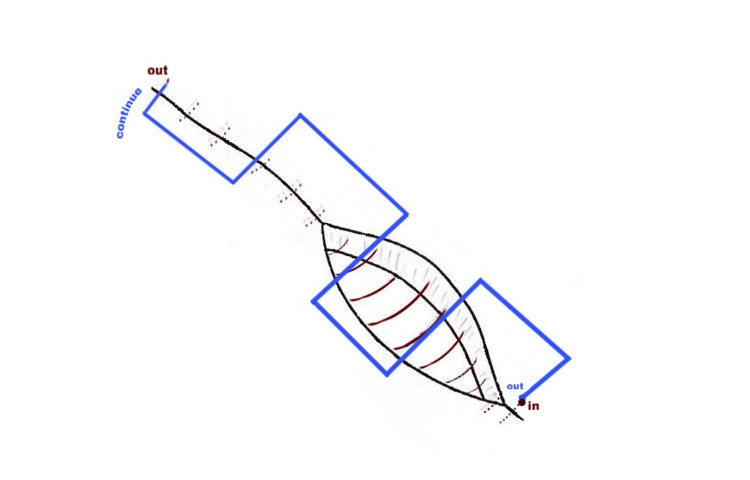
Technique of parallel-layer closure of the uterine incision. A nonlocking technique was used with Vicryl for the first layer. The suture was not tied but was locked in the opposite corner and imbricating sutures were employed with the final knot being tied at the initial corner.

A modification of the purse string suture which we coined “parallel closure” was used in patients during the second period (Figure 2). The same nonlocking technique was used for the first layer but the suture was locked in the opposite corner and imbricating sutures in a parallel manner were employed with the final knot being tied at the initial corner. The hypothesis behind the technique was to support both the superior and inferior superficial myometrial layers to the incision line. Additional sutures were used in the event of continued bleeding from the suture line. Visceral and parietal peritoneum were treated similarly.

**Figure 3:**
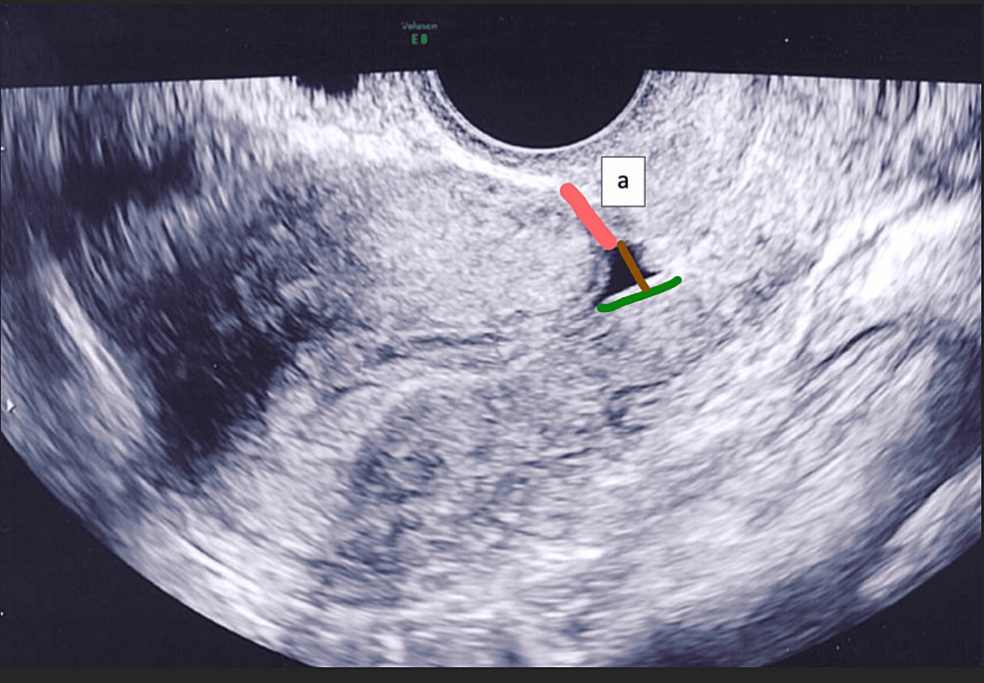
Measurement planes and calculation of isthmocele size. a: residual myometrial thickness.

Ultrasound examinations were performed at 3-6 months and repeated at 18 months postpartum by the primary physician of the patient. The position of the uterus, presence or absence of an isthmocele, and residual myometrial thickness were recorded. Measurements were done according to predefined criteria (Figure 3). Visual data were stored for later review in all patients. All patients were questioned regarding menstrual cycle disturbances at the 18-month follow-up examination. Prolonged bleeding lasting more than five days in the form of spotting, intermenstrual spotting, and heavy menstrual bleeding followed by brownish discharge were regarded as abnormal.

We defined an indentation >2 mm in size at the cesarean section scar site as an isthmocele [13]. Residual myometrial thickness was measured at the site of the cesarean section scar perpendicular to the endometrial stripe. When an isthmocele was present, the distance from the tip of the isthmocele to the serosa was used. All measurements were performed using transvaginal ultrasound (GE Voluson E8 Endocavity Volume Probe RIC5-9-D Real-time 4D micro-convex endocavitary transducer 4-9 MHz) with an empty bladder.

The data analysis was performed using SPSS version 26.0 (IBM Corp., Armonk, NY, USA). To evaluate the patient data, descriptive statistical methods (mean, frequency, and percentage) were used. Categorical variables and continuous variables were calculated as frequency (percentage), mean ± standard deviation (SD), and standard error of the mean (SE) for descriptive statistical analyses. Comparative analysis of residual myometrial thickness by suture layer, niche presence, position of the uterus, and timing of cesarean section was done using independent samples t-test. Categorical variables were analyzed using chi-square and Fisher’s exact tests with Yates post-hoc correction where applicable. A p-value <0.05 was considered statistically significant.

## Results

The patients in the parallel-layer closure group were significantly older than the patients in the single-layer closure group (Table 1). Significantly fewer patients in the parallel-layer closure group presented with an isthmocele both at 3-6 and 18 months postpartum. Moreover, residual myometrium was significantly thicker in the parallel-layer closure group (Table 2). When assessed at 18 months postpartum, patients with a parallel-layer closure had fewer bleeding symptoms that may be attributed to the presence of the isthmocele (Table 2).

**Table 1:** Characteristics of the patients.

	Single-layer closure	Parallel-layer closure	P-value
Patients	295 (59.4%)	202 (40.6%)	
Mean age (years)	34.10	35.36	0.001
Body mass index (kg/m^2^)	22.01	21.9	0.782

**Table 2:** Isthmocele presence, RMT, and the position of the uterus by the closure method. ª: Patients who were available for follow-up at this period. RMT = Residual myometrial thickness; AV = anteverted; RV = retroverted

	Single-layer closure		Parallel-layer closure		P-value
Isthmocele at 3-6 months postpartum	40 (13.6%)		14 (6.9%)		0.029
Isthmocele at 18 months postpartum	48 (16.3%)		16 (7.9%)		0.009
RMT at 3-6 months postpartum (mm)	8.0 ± 2.6		13.2 ± 2.8		0.000
RMT at 18 months postpartum	7.2 ± 2.7		12.3 ± 2.7		0.004
Position of the uterus at 3-6 months postpartum	AV: 234 (79.3%)	RV: 61 (20.7%)	AV: 161 (79.7%)	RV: 41 (20.3%)	0.918
Position of the uterus at 18 months postpartum	AV: 244 (82.7%)	RV: 51 (17.3%)	AV: 172 (85.1%)	RV: 30 (14.1%)	0.470
Symptomatic at 18 months	23 (7.8%) ª		3 (1.5%) ª		0.004

The position of the uterus at 3-6 months and 18 months postpartum follow-up did not differ according to the closure technique (Table 2). When both closure techniques were analyzed together, we found a significant relationship between the position of the postpartum uterus and the frequency of an isthmocele. A retroverted position of the uterus at the 3-6-month follow-up significantly increased the frequency of isthmocele (36/395 (9.1%) with an anteverted uterus and 18/102 (17.6%) with a retroverted uterus; p = 0.022). There was no isthmocele when the uterus was retroverted in the parallel-layer closure group. In patients with a single-layer closure, a retroverted uterus at the 3-6-month follow-up was associated with an isthmocele in 29.5% (18/61) of patients, while no isthmocele was recorded when the uterus was retroverted in the parallel-layer closure group (0/41) (p < 0.001).

Residual myometrial thickness at 3-6 months and 18 months postpartum was significantly greater in patients with an anteverted uterus (p = 0.002 and 0.005 at 3-6 months and 18 months, respectively) (Table 3).

**Table 3:** RMT by position of the uterus (AV vs. RV). RMT = Residual myometrial thickness; AV = anteverted; RV = retroverted

	Uterine position	N	Mean (mm)	P-value
RMT 3-6 months postpartum	AV	395	10.4 ± 3.8	0.002
	RV	102	9.2 ± 3.2	
RMT 18 months postpartum	AV	395	9.5 ± 3.7	0.005
	RV	102	8.4 ± 3.4	

Of the 64 patients with an isthmocele, 26 (40.6%) were symptomatic at 18 months postpartum follow-up. Of the symptomatic patients, 23 were in the single and three were in the parallel-layer closure group (p = 0.004).

**Figure 4:**
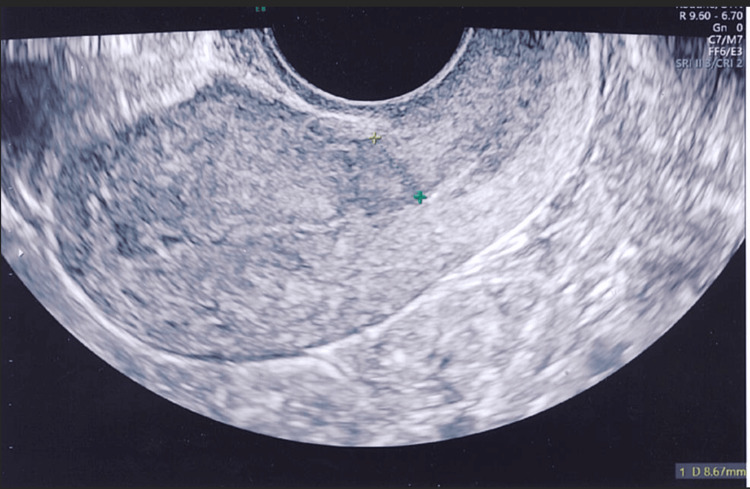
Almost invisible cesarean scar in a patient who underwent parallel closure of lower segment uterine incision (residual myometrial thickness of 8.7 mm).

**Figure 5:**
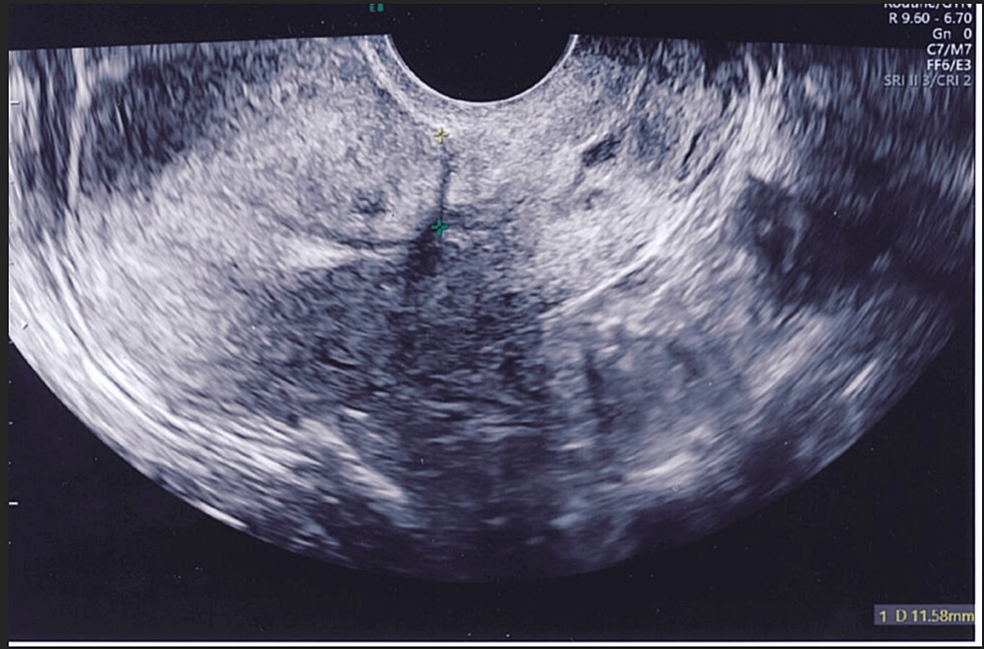
A patient who underwent parallel-layer closure. Postpartum ultrasound shows an anteverted uterus and a visible cesarean scar but no isthmocele (residual myometrial thickness of 11.5 mm).

**Figure 6:**
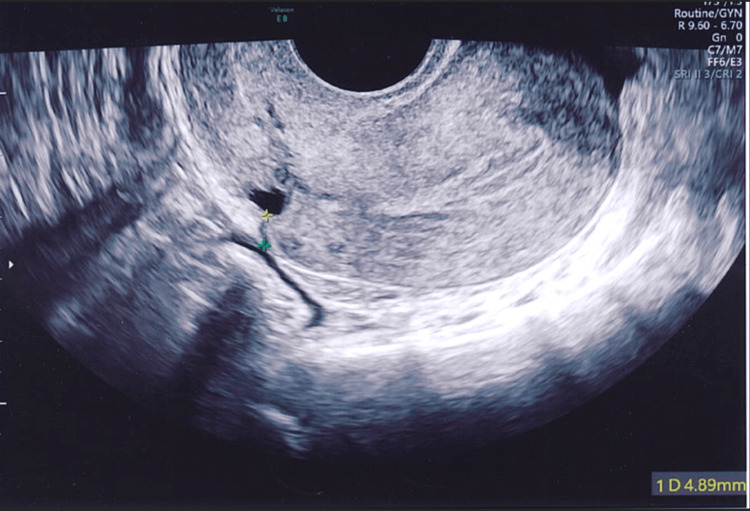
A small isthmocele and a retroverted uterus in a patient who underwent single-layer closure of the lower segment uterine incision (residual myometrial thickness of 4.9 mm).

**Figure 7:**
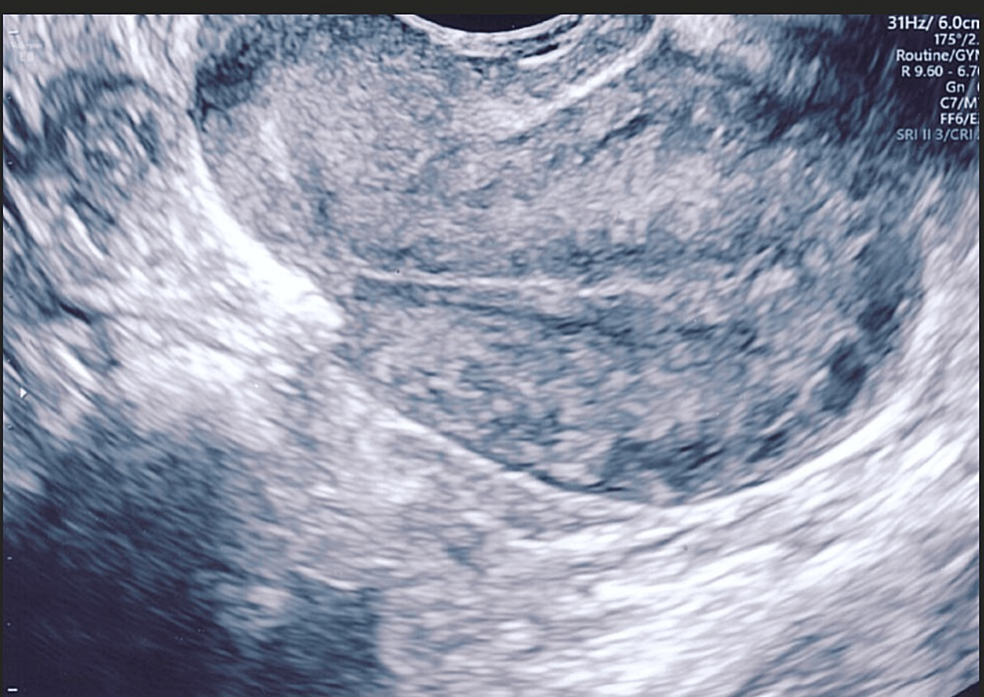
A retroverted uterus and significant thinning of the lower uterine segment in a patient who underwent single-layer closure of the lower segment uterine incision (residual myometrial thickness of 2.5 mm).

**Figure 8:**
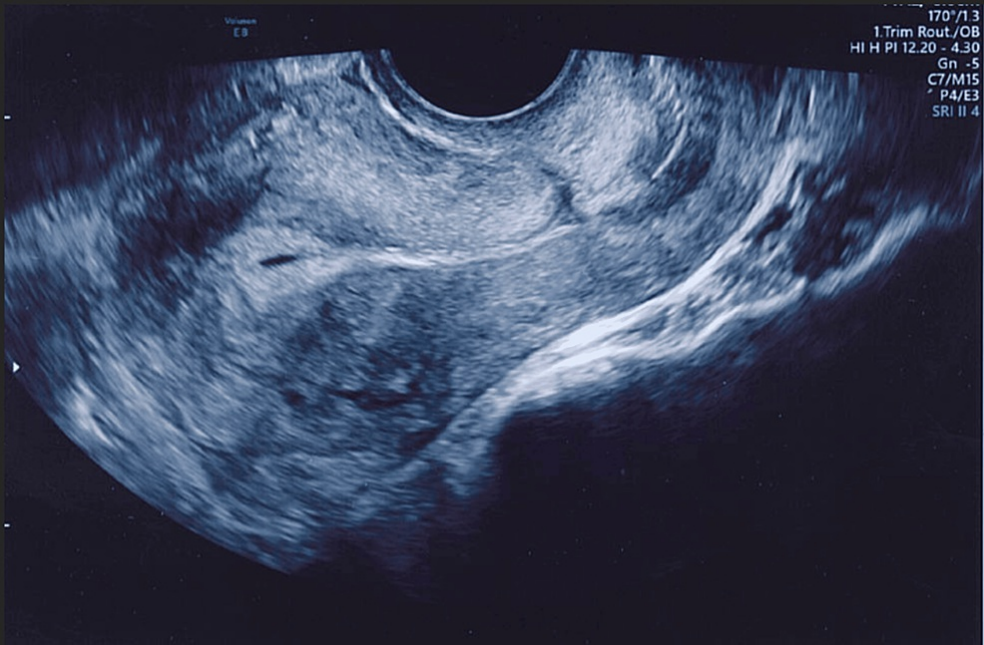
An anteverted uterus with an isthmocele and minimal intracavitary fluid collection in a patient who underwent parallel-layer closure of the lower segment uterine incision (residual myometrial thickness of 4.5 mm).

Figures 4-8 show the ultrasound images of different patients who underwent parallel or single-layer closure of their uterine incisions.

## Discussion

This study shows that a parallel-layer closure of the uterine incision in women undergoing elective cesarean section is associated with a decreased incidence of isthmocele formation and an increased thickness of the residual myometrium compared with a single-layer closure.

It can be speculated that the large postpartum uterus tends to lean backward during the involution phase exerting undue tension on the incision line, thus increasing the risk of inappropriate healing. The novel parallel-layer closure technique differs from other double-layer closure techniques by the placement of the sutures both superior and inferior to the incision line. We implicated this technique as second-layer sutures placed directly on the incision line may cause devascularization of the incision site, thus negatively impacting the wound healing process. With that extra layer of support, the tension on the incision line may be decreased resulting in better healing and a decrease in isthmocele formation together with an increase in the residual myometrial thickness. This is supported by our data that showed a higher incidence of isthmoceles in women with a retroverted uterus compared to those who had an anteverted uterus at 3-6 months postpartum ultrasound (9.1% in anteverted vs. 17.6% in retroverted uteri; p = 0.022). The same was true for residual myometrial thickness (10.4 mm in anteverted vs. 9.2 mm in retroverted uteri; p = 0.002 at 3-6 months postpartum and 9.5 mm in anteverted vs. 8.4 mm in retroverted uteri at 18 months postpartum; p = 0.005) (Table 3). In the parallel-layer closure group, there were no isthmoceles despite the postpartum retroverted position of the uterus. The fact that no isthmoceles were observed in retroverted uteri in the parallel-layer closure group can be explained by the protective effect of the parallel technique against isthmocele formation even in the retroverted position. Despite the relatively small sample size, this is an important finding favoring the parallel-layer technique and can be explained by the theory of reducing the tension on the wound surface.

Two studies found a significant decrease in niche prevalence and niche volume and a thicker residual myometrial thickness after double-layer closure [14,15]. Four randomized controlled trials compared double-layer purse strings with single or double-layer closure in 218 women [16-19]. All studies showed a lower incidence of isthmocele and a thicker residual myometrial layer at the incision site favoring double-layer closure. A relatively small study comparing residual myometrial thickness after double versus single-layer closure using unlocked sutures in both arms and including only women undergoing their first cesarean section was in favor of double-layer closure (6.1 ± 2.2 mm vs. 3.8 ± 1.6 mm; p < 0.001) [15].

A very large multicenter randomized controlled trial that included 2,292 patients compared double-layer versus single-layer closure using nonlocking sutures in women undergoing their first cesarean section showed a significant difference of 4.7% in the incidence of scar defects >2 mm in depth favoring single-layer closure [10]. In this study, isthmoceles were defined as having a depth of at least 2 mm evaluated with ultrasound three months after surgery and reported in 68.9% after single-layer closure and 73.6% after double-layer closure. The mean number of postmenstrual spotting days was 1.33 after single-layer closure and 1.26 after double-layer closure. In this study, double-layer closure was not superior in terms of isthmocele prevalence and short-term menstrual outcomes. Furthermore, there was no difference in long-term (three years) obstetric outcomes, including the rate of patients undergoing a subsequent trial of labor and risk of uterine rupture, nor were there any differences in the number of patients complaining of menstrual disturbances [11]. The investigators concluded that both closure techniques can be used, and the choice of technique should be left to physician preference. However, we do not know which double-layer suture technique was used in this study. In double-layer closure cases, excessive tightening of sutures can compromise blood flow to the incision site, which can impede proper wound healing.

Residual myometrial thickness is a measurable and reproducible entity and is an important criterion for the planning of vaginal delivery after cesarean section [20]. The risk of uterine rupture after a trial of labor is estimated to be 0.7% after one and 1.6% after two previous cesarean sections [21]. Ruptures may involve the anterior or the lateral wall and may cause significant morbidity [22]. In our study, residual myometrium was found to be significantly thicker with the parallel-layer technique compared to the single-layer closure (8.0 mm vs 13.2 mm in single and parallel-layer closure groups, respectively; p = 0.000) (Table 2).

Spotting is an important reason for referral to the gynecologist, bothering many reproductive-aged women. In our study cohort, we evaluated this symptom at 18 months postpartum. Of those who had isthmocele, 39.2% were symptomatic (p = 0.000). An isthmocele was detected in 96% of patients with spotting symptoms. We can conclude that spotting was significantly associated with the presence of isthmocele.

Finally, there is the problem of secondary infertility that needs to be addressed when considering the importance of avoiding isthmocele formation. A recent systematic review that analyzed over 10,000 embryo transfer cycles in women undergoing IVF showed significantly decreased live birth rates in women with an isthmocele compared to women who had a previous cesarean section but without an isthmocele [6]. Women with isthmocele showed a lower live birth rate compared to both women with a previous cesarean section without an isthmocele (adjusted odds ratio (aOR) = 0.62 [95% confidence interval (CI) = 0.53-0.72]) and those with a history of vaginal delivery (aOR = 0.55 [95% CI = 0.42-0.71]).

The strengths of the conclusions can be attributed to the inclusion of all patients in whom the same technique was used by two operators working in the same institution. Our parallel-layer closure technique supports both the superior and inferior superficial myometrial layers to the incision line without interfering with wound vascularization. The two periods were close to each other and there was no difference in the choice of suture materials and closure of the first layer. The exclusion of secondary cesarean cases in the cohort made it possible to avoid the confounding effect of the previous cesarean sections on isthmocele formation. Weaknesses of the study include the nonrandomized design and the nonblinding of the physicians who performed the postpartum ultrasound measurements. However, the availability of visual recordings in all patients may somewhat diminish the impact of the latter. The relatively small number of patients not available for long-term follow-up is another limitation of the study.

## Conclusions

Parallel-layer closure of the uterine incision in women undergoing cesarean section is associated with a decreased incidence of isthmocele formation and an increased residual myometrial thickness compared with a single-layer closure. This may be advantageous in decreasing symptoms associated with an isthmocele and long-term consequences, including scar pregnancy, secondary infertility, and decreased IVF performance. It may also prevent morbidity related to uterine rupture in patients contemplating vaginal birth after cesarean section.

## References

[1] Betran AP, Ye J, Moller AB, Souza JP, Zhang J (2021). Trends and projections of caesarean section rates: global and regional estimates. BMJ Glob Health.

[2] Jordans IP, de Leeuw RA, Stegwee SI (2019). Sonographic examination of uterine niche in non-pregnant women: a modified Delphi procedure. Ultrasound Obstet Gynecol.

[3] Timor-Tritsch IE, D'Antonio F, Calí G, Palacios-Jaraquemada J, Meyer J, Monteagudo A (2019). Early first-trimester transvaginal ultrasound is indicated in pregnancy after previous cesarean delivery: should it be mandatory?. Ultrasound Obstet Gynecol.

[4] Verberkt C, Lemmers M, de Vries R, Stegwee SI, de Leeuw RA, Huirne JA (2023). Aetiology, risk factors and preventive strategies for niche development: a review. Best Pract Res Clin Obstet Gynaecol.

[5] Mohr-Sasson A, Dadon T, Brandt A (2023). The association between uterine scar defect (niche) and the presence of symptoms. Reprod Biomed Online.

[6] Vitagliano A, Cicinelli E, Viganò P (2024). Isthmocele, not cesarean section per se, reduces in vitro fertilization success: a systematic review and meta-analysis of over 10,000 embryo transfer cycles. Fertil Steril.

[7] Hosseini R, Mansoorli S, Pirjani R, Eslamian L, Rabiee M (2021). A comparison of the effects of two suture materials on isthmocele formation: a cohort study. J Gynecol Obstet Hum Reprod.

[8] Ceci O, Cantatore C, Scioscia M, Nardelli C, Ravi M, Vimercati A, Bettocchi S (2012). Ultrasonographic and hysteroscopic outcomes of uterine scar healing after cesarean section: comparison of two types of single-layer suture. J Obstet Gynaecol Res.

[9] Yazicioglu F, Gökdogan A, Kelekci S, Aygün M, Savan K (2006). Incomplete healing of the uterine incision after caesarean section: is it preventable?. Eur J Obstet Gynecol Reprod Biol.

[10] Stegwee SI, van der Voet LF, Ben AJ (2021). Effect of single- versus double-layer uterine closure during caesarean section on postmenstrual spotting (2Close): multicentre, double-blind, randomised controlled superiority trial. BJOG.

[11] Verberkt C, Stegwee SI, Van der Voet LF (2023). Single-layer vs double-layer uterine closure during cesarean delivery: 3-year follow-up of a randomized controlled trial (2Close study) [in press]. Am J Obstet Gynecol.

[12] Caughey AB, Wood SL, Macones GA (2018). Guidelines for intraoperative care in cesarean delivery: Enhanced Recovery After Surgery Society Recommendations (Part 2). Am J Obstet Gynecol.

[13] Vervoort AJ, Uittenbogaard LB, Hehenkamp WJ, Brölmann HA, Mol BW, Huirne JA (2015). Why do niches develop in caesarean uterine scars? Hypotheses on the aetiology of niche development. Hum Reprod.

[14] Hanacek J, Vojtech J, Urbankova I, Krcmar M, Křepelka P, Feyereisl J, Krofta L (2020). Ultrasound cesarean scar assessment one year postpartum in relation to one- or two-layer uterine suture closure. Acta Obstet Gynecol Scand.

[15] Roberge S, Demers S, Girard M (2016). Impact of uterine closure on residual myometrial thickness after cesarean: a randomized controlled trial. Am J Obstet Gynecol.

[16] Dimassi K, Ami O, Merai R, Velemir L, Simon B, Fauck D, Triki A (2022). Double-layered purse string uterine suture compared with single-layer continuous uterine suture: a randomized Controlled trial. J Gynecol Obstet Hum Reprod.

[17] Elkhouly NI, Abdelaal NK, Solyman AE, Elkelani OA, Elbasueny BF, Elhalaby AF (2022). A new technique for uterine incision closure at the time of cesarean section: does it make a difference?. J Obstet Gynaecol.

[18] Heraiz AI, Ibrahem MA, Hamed B (2022). Sonohysterographic evaluation of cesarean scar defect after purse-string versus double-layer uterine closure techniques: a randomized controlled trial. Egyptian J Hosp Med.

[19] Turan C, Büyükbayrak EE, Yilmaz AO, Karsidag YK, Pirimoglu M (2015). Purse-string double-layer closure: a novel technique for repairing the uterine incision during cesarean section. J Obstet Gynaecol Res.

[20] Naji O, Daemen A, Smith A (2013). Changes in cesarean section scar dimensions during pregnancy: a prospective longitudinal study. Ultrasound Obstet Gynecol.

[21] Tanos V, Toney ZA (2019). Uterine scar rupture - prediction, prevention, diagnosis, and management. Best Pract Res Clin Obstet Gynaecol.

[22] Al Naimi A, Mouzakiti N, Wolnicki B, Louwen F, Bahlmann F (2021). Assessing lateral uterine wall defects and residual myometrial thickness after cesarean section. Eur J Obstet Gynecol Reprod Biol.

